# Supplementary material for: From sequence to enzyme mechanism using multi-label machine learning
Source: BMC Bioinformatics. 2014 May 19;15:150. doi: 10.1186/1471-2105-15-150 (PMC4229970; doi:10.1186/1471-2105-15-150)
Supplement: Additional file 2 — Java code of ml2db. Additional file ml2db_code.tar.gz contains the Java source code to run the multi-label machine learning experiments and save the results to database. The code’s Javadoc is included. [file 1471-2105-15-150-S2.zip › additional file 2/ml2db/ml2db/doc/mulan/evaluation/measure/package-frame.html]

mulan.evaluation.measure


# mulan.evaluation.measure

## Interfaces

- *MacroAverageMeasure*
- *Measure*

## Classes

- AveragePrecision
- BipartitionMeasureBase
- ConfidenceMeasureBase
- Coverage
- ErrorSetSize
- ExampleBasedAccuracy
- ExampleBasedBipartitionMeasureBase
- ExampleBasedFMeasure
- ExampleBasedPrecision
- ExampleBasedRecall
- ExampleBasedSpecificity
- GeometricMeanAverageInterpolatedPrecision
- GeometricMeanAveragePrecision
- HammingLoss
- HierarchicalLoss
- InformationRetrievalMeasures
- IsError
- LabelBasedAUC
- LabelBasedAveragePrecision
- LabelBasedBipartitionMeasureBase
- LabelBasedFMeasure
- LabelBasedPrecision
- LabelBasedRecall
- LabelBasedSpecificity
- LossBasedBipartitionMeasureBase
- LossBasedRankingMeasureBase
- MacroAUC
- MacroFMeasure
- MacroPrecision
- MacroRecall
- MacroSpecificity
- MeanAverageInterpolatedPrecision
- MeanAveragePrecision
- MeasureBase
- MicroAUC
- MicroFMeasure
- MicroPrecision
- MicroRecall
- MicroSpecificity
- OneError
- RankingLoss
- RankingMeasureBase
- SubsetAccuracy
